# Maternal antibiotic treatment affects offspring gastric sensing for umami taste and ghrelin regulation in the pig

**DOI:** 10.1186/s40104-021-00557-3

**Published:** 2021-03-18

**Authors:** P. Trevisi, D. Luise, F. Correa, S. Messori, M. Mazzoni, J. P. Lallès, P. Bosi

**Affiliations:** 1 Department of Agricultural and Food Sciences, University of Bologna, Viale G. Fanin 46, 40127 Bologna, Italy; 2 Present Address: World Organisation for Animal Health (OIE), Scientific Secretariat for the STAR-IDAZ International Research Consortium on animal health, 12 rue de Prony, 75017 Paris, France; 3 Department of Veterinary Sciences, University of Bologna, Via Tolara di Sopra, Ozzano nell’Emilia (BO), Bologna, Italy; 4 INRAE, Human Nutrition Division, Site of Theix, 63122 Saint-Genès-Champanelle, France

**Keywords:** Enteroendocrine cell, Gastrin, Ghrelin, Parietal cell, Stomach, Taste receptor

## Abstract

**Background:**

Scarce is knowledge on the process regulating the development of acid secretion, orexigenic signaling, and chemosensing in the stomach of young pigs. Changes of early microbial encounters by suckling pigs can interact with the gut maturation, by the induction of different molecular signaling. Our goal was to assess if the age of offspring and the maternal environment, influenced by sow antibiotic treatment peripartum, could affect gastric morphology and the expression of genes involved in the control of hydrochloric secretion, feed intake, taste, and inflammation in offspring stomach.

**Methods:**

84 pigs from sows fed a diet with amoxicillin (on –d10 to +d21 from farrowing, ANT) or without (CON) were sacrificed at d14, d21, d28 (weaning) or d42. Samples of oxyntic (OXY), pyloric (PY) and cardiac mucosae close to OXY were collected and parietal and enteroendocrine cells (EECs) were counted. Relative gene expression of a set of 11 key genes (*ATP4A*, *SSTR2*, *GAST, GHRL, MBOAT4, PCSK1, GNAT1, TAS1R1, TAS1R3, IL8* and *TNF*) was assessed by qRT-PCR. In addition, 40 offspring obtained from the same ANT and CON sows were offered a normal or a fat-enriched diet for 4 weeks between 140 and 169 d of age, and then OXY and PY were sampled.

**Results:**

The number of parietal and EECs increased with age (*P* < 0.001). *ATP4A* increased with age (within suckling, *P* = 0.043, post-weaning vs. suckling, *P* < 0.001), SSTR2 increased only after weaning (*P* < 0.001). In OXY, *GHRL* increased during suckling (*P* = 0.012), and post-weaning as a trend (*P* = 0.088). *MBOAT4* tended to increase during suckling (*P* = 0.062). *TAS1R1* increased from suckling to post-weaning period (*P* =0.001) and was lower in ANT offspring (*P* = 0.013). *GNAT1* in PY was higher in ANT offspring (*P* = 0.041). Antibiotic treatment of sows peripartum increased expression of *GHRL* and *MBOAT4* in OXY of growing-finishing offspring aged 5 months.

**Conclusions:**

Data show that sensing for umami taste and ghrelin regulation can be affected by maternal environment, but the development of acid secretion, orexigenic signaling and taste perception in the stomach are mostly developmentally controlled.

**Supplementary Information:**

The online version contains supplementary material available at 10.1186/s40104-021-00557-3.

## Background

Like other mammals, pig neonates are exposed to microbes present in the environment, including on the sow, in milk, and on the floor of crates and dejections, and their early intestinal microbiome reflects these contributions [1–3]. Bacterial colonization of the surfaces of the digestive system takes place with the simultaneous increase of signals arising from colostrum intake and digestion [2]. The relevance of the sampling of bacterial motifs and products by host epithelial and specialized cells for the maturation of the local mucosa-associated lymphoid system is already elucidated [3]. Conversely, the interest for the impact of molecules originating from the interaction of bacteria and diet on other signals, for instance, butyrate [4, 5], indole [6] and its metabolites [7], glutamate and structural homologue of ghrelin [8], 3- and 4- methyl-valeric acids, nonanoic acid [4], is only recent. The stimulatory importance of microbes on intestinal stem cells to differentiate to enteroendocrine cells (EECs) was also evidenced [9]. Ghrelin is a hormone produced mainly by EEC in the gastric oxyntic mucosa and in pancreas, and stimulates the appetite. Microbial colonization is necessary to optimize ghrelin production (compared to germ-free condition [10]). This led to the hypothesis that variation in the gastrointestinal microbiome can affect ghrelin expression, explored in the review of Schalla and Stengel [11]. In turn, by triggering the growth hormone secretagogue receptor in neurons of the myenteric plexus throughout the gastrointestinal tract, ghrelin stimulates gastric emptying and increase motility throughout [12]. Thus, ghrelin can be the link between gut microbiome and the control of appetite and gastrointestinal motility.

Oral antibiotics change gut microbiota composition in the porcine small and large intestine [13, 14] in different ways according to the antibiotic type (in feces, [15]). The direct treatment with an antibiotic at precocious time can have a long-lasting effect on the composition and diversity of gut microbiota [16, 17] and reduce the expression of many immune-related processes [18]. Furthermore, the induced microbial disturbances can later dysregulate glucose metabolism in pigs, through e.g. altered pancreatic islet development [19].

Antibiotic use in the feed of pre- and post-farrowing sows can change the piglet micro-environment and can be also an experimental tool to explore mechanisms speeding up or slowing down the development process of the gut function in the young animal [20], with also some long-term physiological impacts [21, 22]. As far as the pig species is concerned, a better knowledge of mechanisms underlying gut development is welcome to better design rearing practices and prepare a more mature pig for the weaning and the whole productive career. Furthermore, antibiotics are used in gestating women for antimicrobial prophylaxis, such as for the prevention of preterm premature rupture of the membranes [23] or perinatal group B streptococcal disease [24]. This practice however can eventually induce dysregulation of the microbiome of the babies [25], but a direct effect of maternal antibiotic treatment on their gastrointestinal functional maturation could not be assessed.

Unlike in human medicine, the stomach is scarcely considered in studies on pig physiology, notwithstanding its relevant contribution to limit the entry of pathogenic microorganisms into the gastrointestinal tract, activate digestion, modulate the passage rate, and start several metabolic controls. This may lead to underestimate its relevance when different feeding strategies are compared. It can be particularly relevant in the fine evaluation of the impact of feeding strategies on digestive function and whole animal metabolism the stomach integrates neural, hormonal, paracrine signals, as well as information’s from the lumen (chemicals and nutrients, xenobiotic components and products) [26]. This implies complex functional relationships with the other segments of the digestive tracts and also involves, in a partial compartmentalization, different gastric areas, as evidenced also for pig [27]. The timeline of gut microbiota sensors, such as toll-like receptors, and of the ability to secrete polymeric immunoglobulin A into the gastric lumen, as shown by the expression of the gene for polymeric immunoglobulin receptor (*PIGR*), was already assessed for piglet [28]. Conversely, less is known about pre- and post-weaning changes in the expression of several genes involved in the control of hydrochloric acid secretion and of eating and taste, although the rapid onset of gastric acid secretion in the first days of pig’s life has long been evidenced [29]. Furthermore, it is not known how much the gastric system of suckling pig exposed to potential novel antigens and dietary stimuli associated with feeding antibiotics to the mother can deviate from the normal physiological development of all the typical gastric functions. On the other hand, less is still known about the impact of a deviation from the standard association of the mother environment with the offspring’s gut microbiota on gastric function in the later productive stages of the pig. This could also be relevant to provide knowledge on the early effects of neonatal environment on gastric functions and metabolic diseases, considering that the pig is an accredited experimental model for studies on human nutritional physiology [30].

For the present work, it was assumed that antibiotic supplementation of sows could affect the microbiological environment of piglets by the contact with the sow excreta, the piglet gut microbiota and, consequently, its gut maturation. Thus, the present work was designed to acquire data on littermates from sows supplemented with an oral antibiotic (amoxicillin) or not during late gestation and lactation, sacrificed during the suckling period or in the post-weaning, with the aim to assess the evolution of integration between sensing of gastric luminal factors, acid secretion, enteroendocrine control and immune activation in the stomach of the young pig. A second aim was to assess if the antibiotic treatment of the mother could extend an effect on the function of the gastric mucosa in the late growth phase of pigs when fed a control diet or a fat-supplemented diet.

## Materials and methods

### Experimental procedure

Twenty-four crossbred (Large White × Landrace) sows from INRA experimental herd, inseminated with Pietrain semen, were used in two successive batches, taking into account parity and resistance of selected fecal bacteria to amoxicillin. Sows were assigned half to the antibiotic group (ANT) and half to the control group (CON), and located per group in different rooms, adopting measures to minimize bacterial cross-contamination between groups. More details on the selection and allocation of sows have been given in previous papers describing the same trial [23, 28]. ANT sows were daily dosed 40 mg/kg body weight of a broad-spectrum antibiotic, amoxicillin (Vetrimoxin PO containing 10% amoxicillin; CEVA Santé Animale, Loudéac, France), together with their morning meal, from day −10 pre-farrowing to day 21 post-farrowing. Among antibiotics approved for use in pigs, amoxicillin was selected because of its large bactericidal spectrum and wide use for group therapies in pigs and poultry [31] and its frequent use in humans, including babies and pregnant women [24]. In addition, amoxicillin has a limited gastrointestinal absorption at therapeutic dose because of its saturable transport [32], therefore, it was supposed to have little direct systemic effects (including in offspring during the period of lactation when the sows of the ANT group received the antibiotic). This was confirmed by recent data on amoxicillin in sow’s milk (very low levels) [20].

A total of 84 piglets, balanced as far as possible by sow and treatment, body weight and gender, were randomly slaughtered at 14, 21 and 28 d of age (23, 23 and 19 pigs, respectively), or at 42 d of age (14 d post-weaning; 19 pigs). For the post-weaning period, litters were kept separated from each other and piglets received a standard weaning diet without addition of antibiotics or other antimicrobial additives. This, in the following text, will be considered as the short-term experiment (STE).

In addition, a total of 10 homogeneous pairs of males or females obtained from CON or ANT sows were maintained to grow selected within litters and randomly assigned to either a low-fat (LF) or a high-fat (HF) diet, starting at the age of 140 d and lasting until day 169 (long-term experiment, LTE). Palm oil (90 g/kg feed) was added to the LF diet (2% crude fat) to obtain the HF diet.

### Animal slaughter and gastric samples collection

At the planned ages, 1 h after the last meal, the piglets were stunned by electric shock immediately followed by killing by exsanguination. For each pig, a midline abdominal incision was made, and the stomach was gently removed. The stomach was opened along the greater curvature, emptied of its contents, and rinsed with double-distilled water.

Tissue samples were collected in three functionally different sites of the stomach: i) in correspondence to the cardiac mucosa close to the oxyntic mucosa in the lesser curvature**,** ii) in the oxyntic (OXY) mucosa (the proper gastric gland region in the body) and iii) in the pyloric (PY) mucosa (in the antrum). The pigs of LTE were slaughtered on the final day of trial and OXY and PY mucosae were obtained.

For RNA extraction and expression analysis, samples from these three gastric sites were collected, snap-frozen in liquid nitrogen and stored at − 80 °C until analysis. For immunohistochemistry, whole-thickness tissue specimens of about 1 cm^2^ were pinned tightly to balsa wood, fixed in 10% buffered formalin for 24 h, dehydrated in a graded series of ethanol and embedded in paraffin.

### Real-time quantitative PCR

Total RNA was isolated from tissue samples according to Takara Fast Pure kit (Takara Bio, Kusatsu, Japan) protocol. For each sample, 1 μg of RNA was reverse-transcribed using the ImProm-II Reverse Transcription System (Promega, Madison, USA).

Ten key genes were analysed in this study. They were selected on the basis of our previous works on porcine stomach: *ATP4A*, ATPase H^+^/K^+^ transporting subunit alpha; *GAST*, gastrin; *GHRL*, ghrelin and obestatin prepropeptide; *GNAT1*, G protein subunit alpha transducin 1; *IL8*, interleukin 8; *MBOAT4*, membrane bound O-acyltransferase domain containing 4; *PCSK1*, proprotein convertase subtilisin/kexin type 1; *SSTR2*, somatostatin receptor 2; *TAS1R1*, taste 1 receptor member 1; *TAS1R3*, taste 1 receptor member 3; *TNF,* tumor necrosis factor. Specific mRNA abundances were determined by real-time quantitative PCR, performed in a LightCycler Real-Time PCR Systems (Roche Diagnostics, Monza, Italy). The reactions, performed in duplicate were carried out in a 10-μL volume containing about 100 ng of cDNA, 0.5 μmol/L of each primer, and 5 μL of SYBR Premix Ex Taq II (Perfect Real Time, Takara Bio, Japan). Reactions consisted in an initial denaturation step at 95 °C for 30 s and 40 cycles of 95 °C for 5 s and the annealing/extension temperature for 20 s. The primer sequences and annealing/extension temperatures are indicated in Supplementary Table 1. Threshold cycles were converted to mRNA molecules/μL using standard curves. Each amplification specificity was checked by melting curve analysis at the end of the reaction. The expression data were normalized by geometric mean of the expression of the two housekeeping genes hydroxymethylbilane synthase and ribosomal protein L4. Primers and amplification conditions for the housekeeping genes are reported in Supplementary Table 1.

### Immunohistochemistry

Paraffin sections (5 μm) of tissue samples underwent immunohistochemical staining for detecting parietal cells in OXY mucosa and EECs in PY mucosa. All the antibodies used in this study are listed in Supplementary Table 2.

Immunostaining of parietal cells was performed as previously reported [33, 34]. Briefly, the sections were treated with 90 mmol/L H_2_O_2_ in methanol for 30 min to block endogenous peroxidase activity and subsequently incubated for 30 min in PBS containing 10% normal goat serum. The sections were then incubated with primary antibody against the H^+^/K^+^-ATPase at 4 °C overnight, by a biotin-conjugated goat anti-mouse IgG and then by ABC complex (Vector Laboratories, Inc., USA). The immune reactions were visualized applying a 3,3′-diaminobenzidine chromogen solution (Vector Laboratories).

Immunostaining of the EECs was performed using the indirect immunofluorescence technique. The sections were incubated at 4 °C overnight in a solution containing chromogranin A primary antibody. After washing in PBS, the sections were incubated for 1 h with goat anti-mouse Alexa Fluor 594 conjugated (Supplementary Table 2).

For each pig, the parietal cells and the EECs were counted in 20 randomly selected glands well oriented perpendicularly to the surface of the mucosa. Cell counting was performed with a 40× objective lens using Nikon Eclipse Ni microscope and the images were recorded with a Nikon DS-Fi2 (for immunohistochemistry) and Nikon DS-Qi1Nc (for immunofluorescence) digital camera and NIS Elements software BR 4.20.01 (Nikon Instruments Europe BV, Amsterdam, the Netherlands). Slight adjustments to contrast and brightness were made using Corel Photo Paint, whereas the figure panels were prepared using Corel Draw (Corel Photo Paint and Corel Draw, Ottawa, ON, Canada).

### Statistical analysis

Data were analyzed using MIXED models System (SAS Institute Inc., Cary, NC, USA).

The effects of treatment (against an error calculated between litters) and time of slaughter (error within litters) for the STE experiment, and the effects of treatment (between litters) and diet (within litters) for the LTE experiment, were tested respectively. The models also included the interaction term between early treatment and age of slaughter (STE) or late diet (LTE). Results are presented as least-squares means and SEM. For time effects, the following three orthogonal contrasts were tested: (1) linear and (2) quadratic effect among the three ages during suckling (14, 21 and 28 d); (3) “Weaned vs. Suckled”, between the post-weaning age (day 42) and the three ages during suckling. Least-squares means comparisons for each combination of treatment and time were made only when a tendency (*P* ≤ 0.10) for an interaction between these terms was observed. Effects were considered significant at *P* ≤ 0.05 and as a trend at *P* ≤ 0.10.

## Results

### Development of gastric acid secretion

**Fig. 1:**
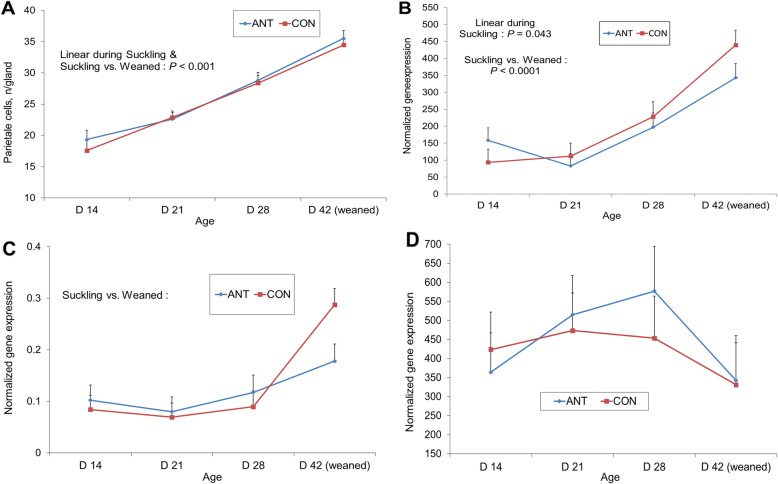
Effect of mother’s antibiotic treatment (CON = control, ANT = antibiotic) and offspring age on parietal cell counts (**a**), ATPase H^+^/K^+^ transporting subunit alpha (*ATP4A*) (**b**) and somatostatin receptor 2 (*SSTR2*) (**c**) expression in oxyntic mucosa and gastrin (*GAST*) (**d**) in pyloric mucosa

The effect of mother’s antibiotic treatment and offspring age on parietal cells count, *ATP4A* and *SSTR2* gene expression in OXY and *GAST* in PY are presented in Fig. 1.

Younger pigs often presented morphological signs indicating the immaturity of OXY (atypical form of the H^+^/K^+^-ATPase-immunoreactive cells, more connective tissue, infiltration of lymphocytes …), and thus 6 pigs of CON and 4 pigs of ANT could not be counted for parietal cells in the first sampling. No effect of the treatment of the mother was seen, nor was any significant interaction with the offspring age. The counts of immunoreactive parietal cells increased with offspring age (within suckling, *P* < 0.001; post-weaning vs. suckling, *P* < 0.001).

Relative gene expression for H^+^/K^+^-ATPase (*ATP4A)* increased also with age (within suckling, *P* = 0.043, post-weaning vs. suckling, *P* < 0.001). *SSTR2* increased only after weaning (*P* < 0.001). In PY, the relative expression for *GAST* was not significantly affected by offspring age.

### Development of sensory taste receptors

**Fig. 2:**
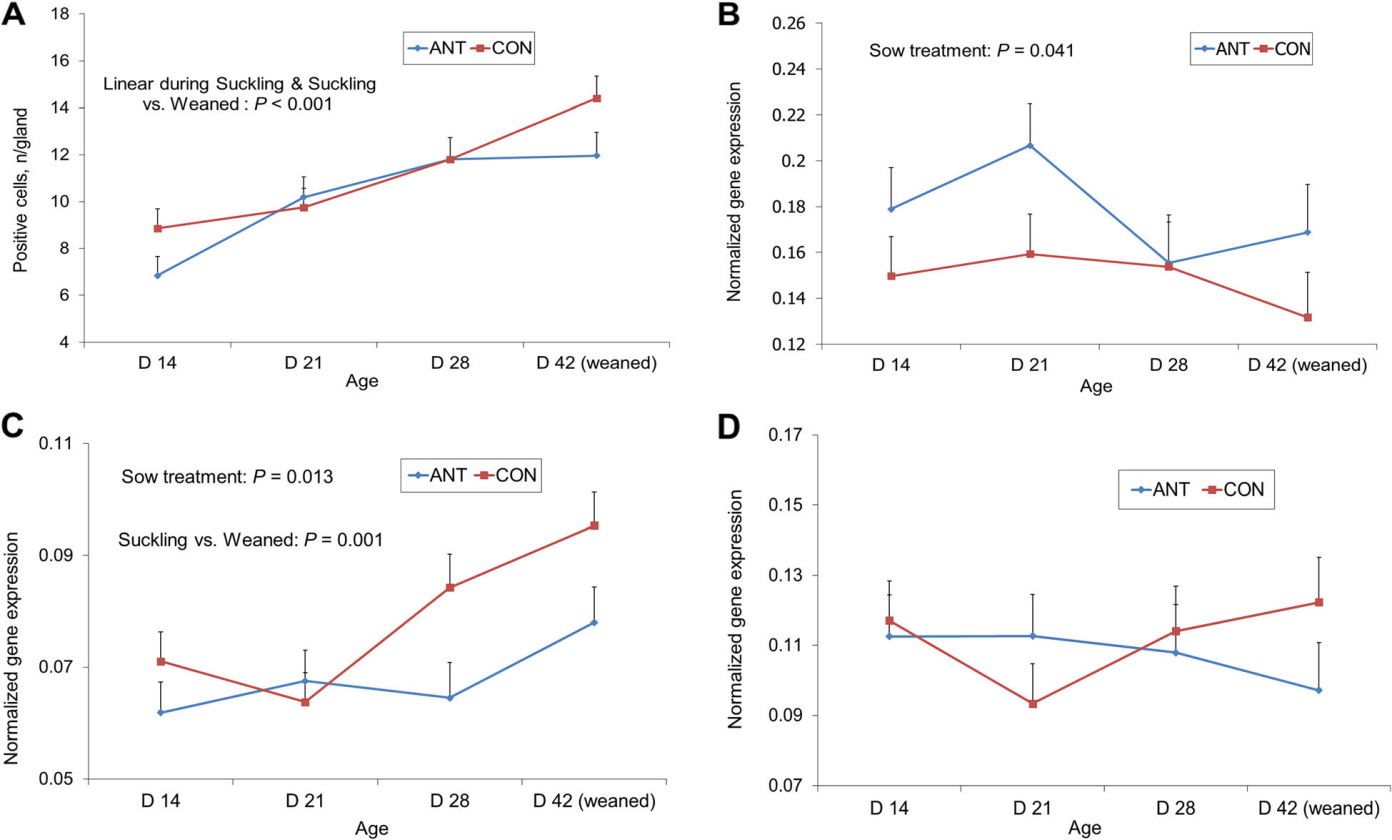
Effect of mother’s antibiotic treatment (CON = control, ANT = antibiotic) and offspring age on enteroendocrine cell count (**a**), G protein subunit alpha transducin 1 (*GNAT1*) (**b**), taste 1 receptor member 1 (*TAS1R*1) (**c**) and taste 1 receptor member 3 (*TAS1R3*) (**d**) expression in pyloric mucosa

The effect of mother’s antibiotic treatment and offspring age on EECs count, *GNAT1*, *TAS1R1* and *TAS1R3*) gene expression in PY mucosa is reported in Fig. 2.

No effect of the interaction of the treatment of the mother with the offspring age was seen. The counts of EECs per each gland, identified by immunohistochemistry for chromogranin A in PY, increased with age (within suckling, *P* = 0.043, post-weaning vs. suckling, *P* < 0.001) and were not influenced by the mother antibiotic treatment. The mother’s treatment reduced the expression of *GNAT1* (*P* = 0.041) and increased that of *TAS1R1* (*P* = 0.013), transcribing the complementary protein for umami sensing*.* Transducin gene expression (*GNAT1*) in PY did not change with age. *TAS1R1* expression increased from suckling to post-weaning period (*P* = 0.001). Expression for *TAS1R3* (necessary for sweet and umami taste) was stable in time. Expression for taste 1 receptor member 1 (complementary for sweet) was not detected.

### Development of orexigenic control

**Fig. 3:**
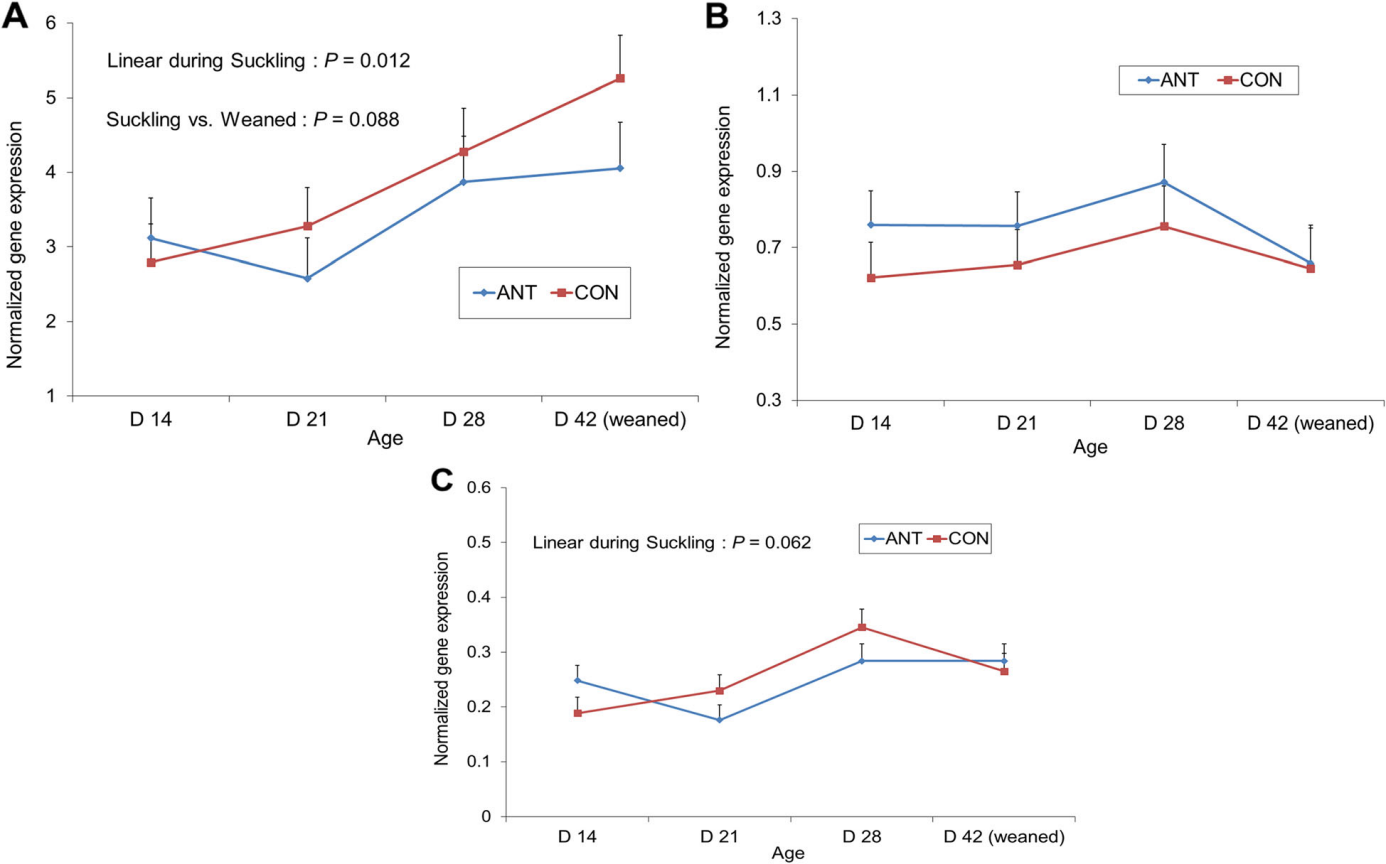
Effect of mother’s antibiotic treatment (CON = control, ANT = antibiotic) and offspring age on expression of ghrelin and obestatin prepropeptide (*GHRL*) (**a**), proprotein convertase subtilisin/kexin type 1 (*PCSK1*) (**b**) and membrane bound O-acyltransferase domain containing 4 (*MPBOAT4*) (**c**) in oxyntic mucosa

The expression of the three genes involved in the gastric release of the active ghrelin (octanoyl-ghrelin): preproghrelin (*GHRL*), proprotein convertase (*PCSK1*), for the posttranslational cleavage, and membrane-bound O-acyltransferase (*MBOAT4*), for acylation of ghrelin, measured in OXY mucosa is presented in Fig. 3. No effect of the treatment of the mother was seen nor a significant interaction with the offspring age. Relative expression of *GHRL* increased during suckling (*P* = 0.012), and also in the post-weaning period as a trend (*P* = 0.088). *MBOAT4* gene expression tended to increase during suckling (*P* = 0.062), while that of *PCSK1* was not affected.

### Development of inflammatory machinery

**Fig. 4:**
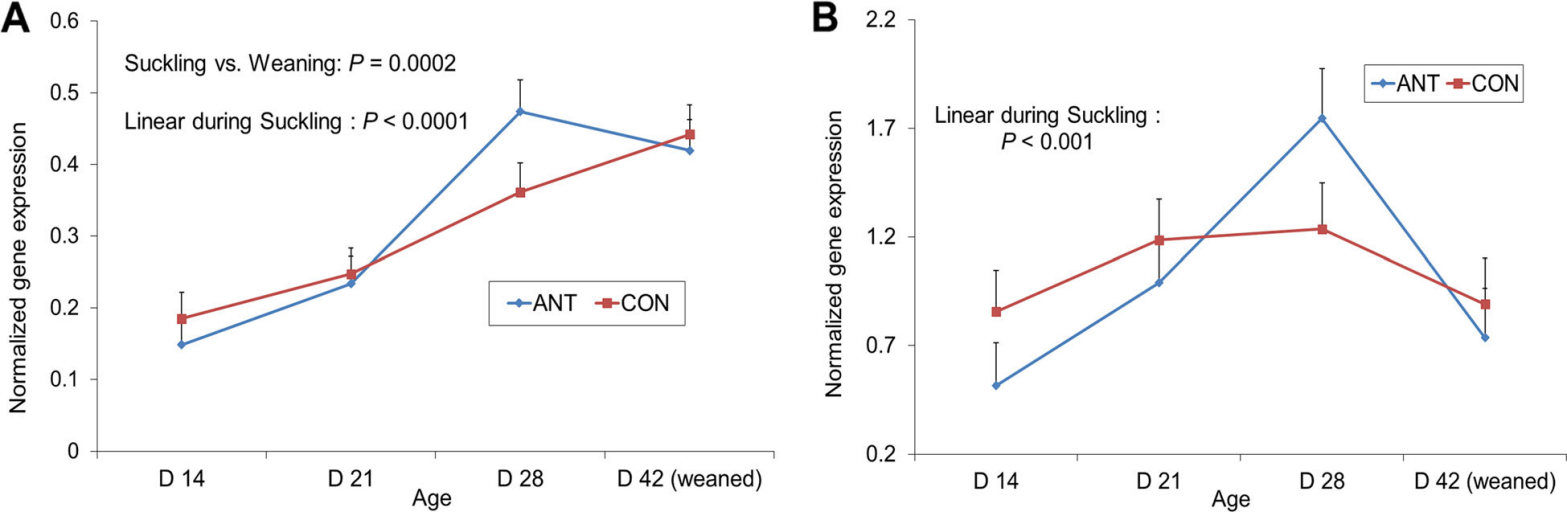
Effect of mother’s antibiotic treatment (CON = control, ANT = antibiotic) and offspring age on gene expression of tumor necrosis factor (*TNF*) (**a**) and interleukin 8 (*IL8*) (**b**) in the transition region between oxyntic and cardia mucosa

The expression of *TNF* and *IL8* genes was also tested in correspondence to the cardiac mucosa close to the oxyntic mucosa in the lesser curvature, where more lymphatic aggregates are seen than in OXY or PY mucosa, in the piglet [35] (Fig. 4). The expression of *TNF* increased linearly (*P* < 0.001) until the post-weaning period, while that of *IL8* increased linearly in the suckling period only (*P* < 0.001).

### Long-term effect on oxyntic and pyloric mucosae

**Fig. 5:**
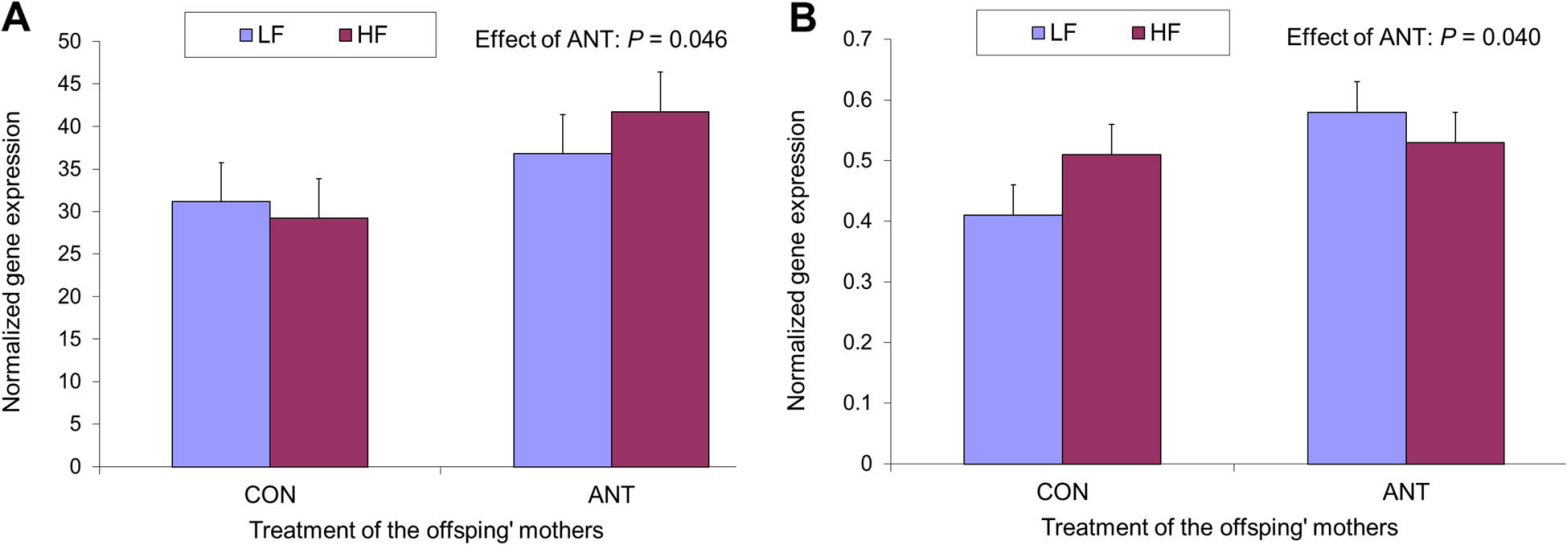
Effect of mother’s antibiotic treatment (CON = control, ANT = antibiotic) and of dietary fat addition in growing-finishing offspring’ diet (LF = low fat, HF = high fat) on gene expression of ghrelin and obestatin prepropeptide (*GHRL*) (**a**) and membrane bound O-acyltransferase domain containing 4 (*MPBOAT4*) (**b**) in oxyntic mucosa of growing pigs

The interaction between the two experimental factors, the treatment of offspring’ mothers and the pigs’ diet, was not significant. The treatment of offspring’ mothers with antibiotic increased the gene expression for *GHRL* and *MBOAT4* in OXY (Fig. 5, *P* < 0.05), but did not affect the expression of *PCSK1* and *ATP4A* in OXY, and *GNAT1, TAS1R1* and *TAS1R3* in PY (data not shown) in growing pigs reared up to 5 months of age*.* No effect of fat addition to the growing pigs’ diet on the expression of any of these genes was seen.

## Discussion

It has already been evidenced that the stomach of the young pig harbors a complex microbiota diffusely distributed throughout the different gastric sectors, differing from the microbiota present in the feed and digesta bolus [36]. This stimulates immune cell recruitment and maturation of the local gastric organized immune system [35, 37]. Furthermore, the early seeding by a complex microbiota is necessary for the activation of typical genes of the OXY mucosa, as compared with the association with a simplified microbiota [37]. Dietary intervention with amoxicillin on sows in the pre-farrowing days induced changes in their fecal microbial profile [20, 21], with an increase of microbial diversity on day 1 after farrowing, compared to control [20], or a reduction of lactobacilli [21]. In addition a reduced microbial diversity was seen in vaginal swabs collected at farrowing [20]. This, in turn, delayed the morphological maturation of the small intestine of their offspring [20], but without evident effect on the offspring gut microbiota diversity. The delayed intestinal maturation could be related to the age-dependent effect of the mother treatment on the offspring’ microbiota that in previous observations on the same experiment was more affected in the small [21] than in the large intestine [22]. Nevertheless, these variations were not associated to an effect of ANT on newborn body weight, or daily weight gain in the suckling period [20, 21], although ANT increased the number of pigs born alive and reduced the number of stillborn [20]. It is possible also that different quality or quantity of milk production from sows supplemented with amoxicillin could have had equalized the growth offspring response compared to control sows, but no data are available to verify this hypothesis. No analysis on volatile fatty acids present in feces was made. Additionally, we did not investigate such antibiotic effects on the microbiota or volatile fatty acids or other bacterial metabolites present in the stomach of piglets. Therefore, it is hardly possible to speculate presently on the types of possible links between intestinal microbiota in the mother and the effects observed in the stomach of piglets. In the present work, the effect of mother’s treatment was overall limited in the offspring stomach to the expression of two genes out of 11 genes studied: one gene deputed to the testing of bolus in transit (*TAS1R1,* umami taste, upregulated) and the other one in the translation of the detected signals (*GNAT,* transducing, downregulated), respectively, in the pyloric region only. This region is presumed to be more in intimate contact with the content of passing digesta, due to its funnel shape. This may be related to the fact that the gastric microbiota of the piglet rapidly structures in a peculiar way that does not fully resemble the profile of the microbiota present in the passing gastric bolus [36]. To our knowledge, no experimental evidence was reported for a direct association between variations in the gut microbiota and the degree of regulation of umami sensing. However, data from human and *in vitro* studies suggest an involvement of TAS1 receptors in the interplay between bacteria and fungi and the host by the recognition of compounds produced by the microorganisms [38, 39]. Recognition of dietary molecules along the gut is important to activate digestive process, regulate feed intake and transit, and meal duration. This holds true also for the stomach and for the machinery that detects and processes the taste signals. Umami taste detection is in general associated to the presence of glutamic acid that was proposed as a marker molecule used by the stomach to sense the degree of gastric protein digestion (or partial hydrolysis) [40]. This explains the favoring effect of glutamate on antral distension observed in humans [41, 42], that was associated [41] or not [42] with gastric emptying, presumably depending on the experimental dietary conditions. In preterm piglets with partial enteral nutrition, gastric emptying was slowed [43]. The presence or absence of effect of gastric glutamate sensing on gastric emptying could have practical relevance for the potential satiating effect. However, in piglets, the supplementation with glutamate did not affect feed intake [44]. Thus, no evidence is available to suppose that an increased expression of *TAS1R1* could interfere with piglet appetite. Interestingly, it has been observed that typical bacterial metabolites, butyrate and propionate, can increase mRNA levels of *TAS1R1* and *TAS1R3* of intestinal EECs altering their sensitivity to glutamate with a mechanism related to the detection by free fatty acid receptors 2 and 3 [45]. The content of volatile fatty acids in the stomach of our piglets was not measured but it is well known that significant amounts of them can be detected in the gastric content of piglets [46]. This supports the possibility that variations in the gastric microbiome can interfere with pyloric enteroendocrine sensing of dietary signals and that this response can be changed by a transgenerational effect, such as the long memory of antibiotic treatment of the mother [47]. However, further investigations are necessary to support our hypothesis.

Thus, it can be hypothesized that mild variations in the gastric microbial profile or activity could have changed the entity of protein degradation. Along the porcine digestive tract, transducin α-subunit G protein (*GNAT1*) is highly expressed in PY mucosa and the presence of immunoreactive cells for this α-subunit was sharply reduced when young pigs are fasted or after refeeding [48]. Based on these observations, it is tempting to speculate that the reduction of *GNAT1* expression in offspring of antibiotic-treated sows was related to changes in signals associated with feed presence or passage rate in the stomach. However, at least as far as orexigenic response is considered, the absence of effect on *GHRL*, *PCSK1* and *MPBOAT4* in OXY mucosa, does not support this hypothesis.

The observation of an upregulating effect of mothers’ treatment in the LTE on *GHRL* and *MPBOAT4* indicates, interestingly, a programmed regulation early in life. Ghrelin is the only hormone known to promote feed intake and is locally implicated also in upper gastrointestinal tract motility and in gastric secretion. This effect depends on the sensitivity to ghrelin, especially in the acylated form, of the growth hormone secretagogue receptor mainly located in the region of the myenteric plexus and submucosal plexus of the gastrointestinal tract [12]. Unfortunately, the expression of the gene for this receptor in the gastrointestinal mucosa samples is generally very low, for instance in fundic [27, 37] and pyloric [27] areas of growing pigs, and not detectable with our qRT-PCR method. Thus, we could not verify if there was an effect of antibiotic treatment in mothers on the expression of that gene in offspring. Eating behavior has productive relevance and can be represented by emerging traits for swine production including eating time per day or group feed intake [49]. The present observation of the relevance of the origin and the early conditions of piglets for the expression of genes related to the orexigenic control could explain at least partially the variability of such productive traits. However, to our knowledge, no study was addressed to quantify ghrelin (in blood or as gene expression) in growing pigs with different eating behavior. Considering swine as a translational model of human, a hunger-stimulating effect should be considered in the presence of antibiotic-induced early modification of the gut microbiota. This could fit with the suppression of hunger-reducing signal observed along the gut in case of intestinal microbial dysbiosis in human [50]. Furthermore, the upregulation of *MBOAT4* can have negative implications for the gut health, as indirectly suggested by the attenuation of colitis associated with reduced inflammation and improved intestinal tight junction function, when this gene is knockdown in mice [51]. Conversely, the absence of effect of dietary addition of oil in growing-finishing pigs may depend on its moderate addition (9%) that is quite high for the typical pig production but very moderate for a standard human western diet.

Conversely, at least for morphometric evaluations (parietal and EECs) and for the considered genes, it is possible that variations in the maternal environment microbiota were not large enough to induce detectable variations at the considered ages of offspring. Otherwise, considering the variations of the single bacterial genera that were differentially affected by mothers’ treatment in the small intestine, it could be that they were functionally mutually compensating in the different treatment groups. For example, for lactobacilli, that was one of the most abundant genera in the piglet stomach [36], a higher abundance of *Lactobacillus gasseri* and *L. delbrueckii* evidenced in the treated group, was offset by a higher abundance of *L. acidophilus* in the control group.

Regarding differences observed with offspring age, the naturally developmental program of the different gastric functions, supported by the induction of changes of the diet and in the microbiota, was in general confirmed [30, 52, 53]. The relative stability of *GAST* expression in the suckling period agrees with the early observations of Cranwell and Hunsky [54], showing that gastrin in blood serum raises very soon and remains quite stable in the suckling pig. Early activity of gastrin is particularly important for the growth of the stomach [55]. Conversely, Cranwell and Hunsky [54] observed a rise of values in pig fed solid feed. The discrepancy can be presumably related to the concomitant increase of the gastric surface, pylorus included [56]. Also, the stability of SSTR2 during suckling agrees with constant level of somatostatin in the gastric mucosa [57]. SSTR2 mediates the action of somatostatin in parietal cells, EECs, and gastrin-secreting cells [58].

The progressive complexity of required endocrine response before weaning and afterwards is underlined by the increased number of EECs counted in PY mucosa. Indeed, EECs sense molecules in the flowing digesta content, elaborate the signals and respond with the release of an array of signaling molecules, that, depending on what was detected, have different physiological functions, locally (paracrine and/or autocrine), centrally (gut-brain axis), or along the gut [59]. Studies specifically aimed at testing the age effect on the gastric sensing in pigs are not available, but the ability to adapt their number to different nutrients (e.g. butyrate [34]) or to modulate their specific detection ability of microbial metabolites (e.g. OR51E1 receptor [4]) have been already documented in young pigs. Conversely, in the present study, little effect of age within suckling or of weaning was seen for *TAS1R1* and *TAS1R3* genes or for *GANT1*, a gene deputed to signal transduction in EECs. For the first two genes, which transcribe for the two dimers of the umami receptor, the regulatory network in pigs has already been elucidated [60]. However, it is not clear in piglets whether it is finely tuned and how. Nevertheless, glutamate content is abundant in milk and also in several raw materials (e.g. wheat and wheat by-products) (INRA-AFZ tables [61]) used for the pre-starter feed, thus it is reasonable that it does not change sharply.

Finally, concerning inflammatory and immune activation, the constant increase of *TNF* gene expression with age explains the parallel activation of *PIGR* previously reported in the same gastric area [28], because TNF produced by epithelial cells contributes to up-regulate the expression of mRNA for *PIGR* in the same cells [62]. Conversely, the reduction of *IL8* gene expression after weaning may be related to the relative stabilization of the activity of bacteria-responding Toll-like receptor 4 [14], considering that this receptor modulates IL8 through the Fas signaling pathway [63]. Alternatively, it can be hypothesized that immune “tolerance” to bacterial pro-inflammatory products, e.g. lipopolysaccharide, was already acquired [64].

## Conclusions

With the present work we started to provide elements to enlarge knowledge on the development of acid secretion, orexigenic signaling, and taste perception in the stomach of young pigs.

The sensing for umami taste and ghrelin regulation were shown to be affected by maternal environment in the present work, but the development of acid secretion, orexigenic signaling, taste perception, immune response in the stomach are mostly developmentally controlled.

A long-term hunger-stimulating effect based on data of expression of genes for ghrelin and for its transformation observed on growing-finishing pigs should be considered in the presence of antibiotic-induced early modification of the gut microbiota of the pig’s mother.

## Supplementary Information

**Additional file 1: Supplemental Table 1.** Primers information and real-time quantitative PCR conditions used in the trial. **Supplemental Table 2.** Characteristics of antibodies used for immunostaining.

**Additional file 2: Supplementary file 1**. Individual records of STE.

**Additional file 3: Supplementary file 2**. Individual records of LTE.

## Data Availability

All the individual data of all the variables under investigation in this study are included in this published article and its supplementary xlsx files 1 and 2, for STE and LTE, respectively.

## Abbreviations

ANT: Antibiotic group
ATP4A: ATPase H^+^/K^+^ transporting subunit alpha
CON: Control group
EEC: Enteroendocrine cell
GAST: Gastrin
GHRL: Ghrelin and obestatin prepropeptide
GNAT1: G protein subunit alpha transducin 1
HF: High-fat
IL8: Interleukin 8
LF: Low-fat
LTE: Long-term experiment
MBOAT4: Membrane bound O-acyltransferase domain containing 4
OXY: Oxyntic mucosa
PCSK1: Proprotein convertase subtilisin/Kexin type 1
PY: Pyloric mucosa
SSTR2: Somatostatin receptor 2
STE: Short-term experiment
TAS1R1: Taste 1 receptor member 1
TAS1R3: Taste 1 Receptor Member 3
TNF: Tumor Necrosis Factor

## References

[1] Chen W, Mi J, Lv N, Gao J, Cheng J, Wu R (2018). Lactation stage-dependency of the sow milk microbiota. Front Microbiol.

[2] Chen X, Xu J, Ren E, Su Y, Zhu W. Co-occurrence of early gut colonization in neonatal piglets with microbiota in the maternal and surrounding delivery environments. Anaerobe. 2018;49:30–40. 10.1016/j.anaerobe.2017.12.002 29223548

[3] Zhang Z, Kwawukume A, Moossavi S, Sepehri S, Nyachoti M, Khafipour E. Time series and correlation network analyses to identify the role of maternal microbiomes on development of piglet gut microbiome and susceptibility to neonatal porcine diarrhea. J Anim Sci. 2018;96(suppl.2):213.

[4] Priori D, Colombo M, Clavenzani P, Jansman AJ, Lallès JP, Trevisi P (2015). The olfactory receptor OR51E1 is present along the gastrointestinal tract of pigs, co-localizes with enteroendocrine cells and is modulated by intestinal microbiota. PLoS One.

[5] Zhong X, Zhang Z, Wang S, Cao L, Zhou L, Sun A (2019). Microbial-driven butyrate regulates Jejunal homeostasis in piglets during the weaning stage. Front Microbiol.

[6] Chimerel C, Emery E, Summers DK, Keyser U, Gribble FM, Reimann F (2014). Bacterial metabolite indole modulates incretin secretion from intestinal enteroendocrine L cells. Cell Rep.

[7] Hubbard TD, Murray IA, Perdew GH (2015). Indole and tryptophan metabolism: endogenous and dietary routes to ah receptor activation. Drug Metab Dispos.

[8] Mazzoli R, Pessione E (2016). The neuro-endocrinological role of microbial glutamate and GABA signaling. Front Microbiol.

[9] Cani PD, Knauf C (2016). How gut microbes talk to organs: the role of endocrine and nervous routes. Mol Metab.

[10] Weger BD, Gobet C, Yeung J, Martin E, Jimenez S, Betrisey B (2019). The mouse microbiome is required for sex-specific diurnal rhythms of gene expression and metabolism. Cell Metab.

[11] Schalla MA, Stengel A. Effects of microbiome changes on endocrine ghrelin signaling-a systematic review. Peptides. 2020;133:170388. 10.1016/j.peptides.2020.170388 32846187

[12] Sanger GJ, Furness JB (2016). Ghrelin and motilin receptors as drug targets for gastrointestinal disorders. Nat Rev Gastroenterol Hepatol.

[13] Yu M, Mu C, Zhang C, Yang Y, Su Y, Zhu W (2018). Marked response in microbial community and metabolism in the ileum and cecum of suckling piglets after early antibiotics exposure. Front Microbiol.

[14] Zhang C, Peng Y, Mu C, Zhu W (2018). Ileum terminal antibiotic infusion affects jejunal and colonic specific microbial population and immune status in growing pigs. J Anim Sci Biotechno.

[15] Bosi P, Merialdi G, Scandurra S, Messori S, Bardasi L, Nisi I (2011). Feed supplemented with three different antibiotics improved food intake and reduced the activation of the humoral immune response in healthy weaned pigs but had differing effects on intestinal microbiota. J Anim Sci.

[16] Ruczizka U, Metzler-Zebeli B, Unterweger C, Mann E, Schwarz L, Knecht C (2020). Early parenteral administration of ceftiofur has gender-specific short-and long-term effects on the fecal microbiota and growth in pigs from the suckling to growing phase. Animals..

[17] Zhang CJ, Yu M, Yang YX, Mu CL, Su Y, Zhu WY. Effect of early antibiotic intervention on specific bacterial communities and immune parameters in the small intestine of growing pigs fed different protein level diets. Animal. 2020. 10.1017/S1751731120001044. 10.1017/S1751731120001044 32436487

[18] Schokker D, Zhang J, Vastenhouw SA, Heilig HG, Smidt H, Rebel JM (2015). Long-lasting effects of early-life antibiotic treatment and routine animal handling on gut microbiota composition and immune system in pigs. PLoS One.

[19] Li J, Yang K, Ju T, Ho T, McKay CA, Gao Y (2017). Early life antibiotic exposure affects pancreatic islet development and metabolic regulation. Sci Rep.

[20] de Greeff A, Schokker D, Roubos-van den Hil P, Ramaekers P, Vastenhouw SA, Harders F, et al. The Effect of maternal antibiotic use in sows on intestinal development in offspring. J Anim Sci. 2020. 10.1093/jas/skaa181. 10.1093/jas/skaa181 PMC7295330 32479635

[21] Arnal ME, Zhang J, Messori S, Bosi P, Smidt H, Lallès JP (2014). Early changes in microbial colonization selectively modulate intestinal enzymes, but not inducible heat shock proteins in young adult swine. PLoS One.

[22] Arnal ME, Zhang J, Erridge C, Smidt H, Lallès JP (2015). Maternal antibiotic-induced early changes in microbial colonization selectively modulate colonic permeability and inducible heat shock proteins, and digesta concentrations of alkaline phosphatase and TLR-stimulants in swine offspring. PLoS One.

[23] Fitzgibbon A, Clooney L, Broderick D, Eogan M, McCallion N, Drew RJ. Erythromycin compared to amoxicillin and azithromycin for antimicrobial prophylaxis for preterm premature rupture of the membranes: a retrospective study. J Obstet Gynaecol. 2020:1–4, Online ahead of print. 10.1080/01443615.2020.1786806 32799726

[24] Verani JR, McGee L, Schrag SJ (2010). Prevention of perinatal group B streptococcal disease. Morb Mortal Wkly Rep (MMWR).

[25] Mazzola G, Murphy K, Ross RP, Di Gioia D, Biavati B, Corvaglia LT (2016). Early gut microbiota perturbations following intrapartum antibiotic prophylaxis to prevent group B streptococcal disease. PLoS One.

[26] Chu S, Schubert ML (2013). Gastric secretion. Curr Opin Gastroen.

[27] Colombo M, Priori D, Trevisi P, Bosi P (2014). Differential gene expression in the oxyntic and pyloric mucosa of the young pig. PLoS One.

[28] Trevisi P, Gandolfi G, Priori D, Messori S, Colombo M, Mazzoni M (2013). Age-related expression of the polymeric immunoglobulin receptor (pIgR) in the gastric mucosa of young pigs. PLoS One.

[29] Low AG (1990). Nutritional regulation of gastric secretion, digestion and emptying. Nutr Res Rev.

[30] Roura E, Koopmans SJ, Lallès JP, Le Huerou-Luron I, De Jager N, Schuurman T (2016). Critical review evaluating the pig as a model for human nutritional physiology. Nutr Res Rev.

[31] Patyra E, Kwiatek K (2020). In-house validation method for quantification of amoxicillin in medicated feedingstuffs with the use of HPLC-DAD technique. J Vet Res.

[32] Paintaud G, Alvan G, Dahl ML, Grahnen A, Sjövall J, Svensson JO (1992). Nonlinearity of amoxicillin absorption kinetics in human. Eur J Clin Pharmacol.

[33] Bosi P, Mazzoni M, De Filippi S, Casini L, Trevisi P, Petrosino G, Lalatta-Costerbosa G (2006). Continuous dietary supply of free calcium formate negatively affects parietal cell population and gastric RNA expression for H+/K+-ATPase in weaning pigs. J Nutr.

[34] Mazzoni M, Le Gall M, De Filippi S, Minieri L, Trevisi P, Wolinski J (2008). Supplemental sodium butyrate stimulates different gastric cells in weaned pigs. J Nutr.

[35] Mazzoni M, Bosi P, De Sordi N, Giovanna L-CG (2011). Distribution, organization and innervation of gastric MALT in conventional piglet. J Anat.

[36] Motta V, Trevisi P, Bertolini F, Ribani A, Schiavo G, Fontanesi (2017). Exploring gastric bacterial community in young pigs. PloS One.

[37] Trevisi P, Priori D, Motta V, Luise D, Jansman AJ, Koopmans SJ (2017). The effects of starter microbiota and the early life feeding of medium chain triglycerides on the gastric transcriptome profile of 2-or 3-week-old cesarean delivered piglets. J Anim Sci Biotechno..

[38] Barham HP, Cooper SE, Anderson CB, Tizzano M, Kingdom TT, Finger TE (2013). Solitary chemosensory cells and bitter taste receptor signaling in human sinonasal mucosa. Int Forum Allergy Rhinol.

[39] Gulbransen BD, Clapp TR, Finger TE, Kinnamon SC (2008). Nasal solitary chemoreceptor cell responses to bitter and trigeminal stimulants in vitro. J Neurophysiol.

[40] Uneyama H (2011). Nutritional and physiological significance of luminal glutamate-sensing in the gastrointestinal functions. Yakugaku Zasshi.

[41] Zai H, Kusano M, Hosaka H, Shimoyama Y, Nagoshi A, Maeda M (2009). Monosodium l-glutamate added to a high-energy, high-protein liquid diet promotes gastric emptying. Am J Clin Nutr.

[42] Boutry C, Matsumoto H, Airinei G, Benamouzig R, Tomé D, Blachier F (2011). Monosodium glutamate raises antral distension and plasma amino acid after a standard meal in humans. Am J Physiol Gastrointest Liver Physiol.

[43] Bauchart-Thevret C, Stoll B, Benight NM, Olutoye O, Lazar D, Burrin DG (2013). Supplementing monosodium glutamate to partial enteral nutrition slows gastric emptying in preterm pigs. J Nutr.

[44] Amorim AB, Saleh MAD, Miassi GDM, Berto DA (2018). Dietary supplementation with glutamine or glutamic acid for weanling piglets. Pesqui Agropecu Bras.

[45] Shackley M, Brown AJ, Tate EW, Frost G, Hanyaloglu AC. Short chain fatty acids enhance expression and activity of the umami taste receptor in enteroendocrine cells via a Gαi/o pathway. BioRxiv. 2020. 10.1101/2020.06.01.127316. 10.3389/fnut.2020.568991 PMC7658341 33195366

[46] Friend DW, Cunningham HM, Nicholson JWG (1963). The production of organic acids in the pig: ii. The effect of diet on the levels of volatile fatty acids and lactic acid in sections of the alimentary tract. Can J Anim Sci.

[47] Lallès JP, Michel C, Theodorou V, Segain JP. Epigenetic regulation of gastrointestinal epithelial barrier and developmental origins of health and disease. In: Rosenfeld CS, editor. The epigenome and developmental origins of health and disease. Elsevier, Amsterdam, The Netherlans. 2016. pp. 337–360.

[48] Mazzoni M, De Giorgio R, Latorre R, Vallorani C, Bosi P, Trevisi P (2013). Expression and regulation of α-transducin in the pig gastrointestinal tract. J Cell Mol Med.

[49] Aldridge MN, Bergsma R, Calus MPL. Investigating novel traits in single trait selection for their potential in selection indexes for feed efficiency of crossbred pigs. Proc Assoc Advmt Anim Breed Genet. 2019;23:218–21.

[50] Amabebe E, Robert FO, Agbalalah T, Orubu ES (2020). Microbial dysbiosis-induced obesity: role of gut microbiota in homoeostasis of energy metabolism. Brit J Nutr..

[51] Tian P, Lu X, Jin N, Shi J (2020). Knockdown of ghrelin-O-acyltransferase attenuates colitis through the modulation of inflammatory factors and tight junction proteins in the intestinal epithelium. Cell Biol Int.

[52] Guilloteau P, Zabielski R, Hammon HM, Metges CC (2010). Nutritional programming of gastrointestinal tract development. Is the pig a good model for man?. Nut Res Rev.

[53] Odle J, Lin X, Jacobi SK, Kim SW, Stahl CH (2014). The suckling piglet as an agrimedical model for the study of pediatric nutrition and metabolism. Annu Rev Anim Biosci.

[54] Cranwell PD, Hansky J (1980). Serum gastrin in newborn, sucking and weaned pigs. Res Vet Sci.

[55] Håkanson R, Oscarson J, Sundler F (1986). Gastrin and the trophic control of gastric mucosa. Scand J Gastroentero.

[56] Cranwell PD (1985). The development of acid and pepsin (EC 3. 4. 23. 1) Secretory capacity in the pig; the effects of age and weaning: 1. Studies in anaesthetized pigs. Brit J Nutr.

[57] Sangild PT, Cranwell PD, Hilsted L (1992). Ontogeny of gastric function in the pig: acid secretion and the synthesis and secretion of gastrin. Neonatology..

[58] Schubert ML (2010). Gastric secretion. Curr Opin Gastroenterol.

[59] Latorre R, Sternini C, De Giorgio R, Greenwood-Van MB (2016). Enteroendocrine cells: a review of their role in brain–gut communication. Neurogastroent Motil.

[60] Kim JM, Ren D, Reverter A, Roura E (2016). A regulatory gene network related to the porcine umami taste receptor (TAS 1R1/TAS 1R3). Anim Genet.

[61] Sauvant D, Perez J-M, Tran G (2004). Tables of composition and nutritional value of feed materials: pigs, poultry, cattle, sheep, goats, rabbits, horses and fish.

[62] Reséndiz-Albor AA, Reina-Garfias H, Rojas-Hernández S, Jarillo-Luna A, Rivera-Aguilar V, Miliar-García A (2010). Regionalization of pIgR expression in the mucosa of mouse small intestine. Immunol Lett.

[63] Fernandes P, O’Donnell C, Lyons C, Keane J, Regan T, O’Brien S (2014). Intestinal expression of Fas and Fas ligand is upregulated by bacterial signaling through TLR4 and TLR5, with activation of Fas modulating intestinal TLR-mediated inflammation. J Immunol.

[64] Abreu MT, Vora P, Faure E, Thomas LS, Arnold ET, Arditi M (2001). Decreased expression of toll-like receptor-4 and MD-2 correlates with intestinal epithelial cell protection against dysregulated proinflammatory gene expression in response to bacterial lipopolysaccharide. J Immunol.

