# Supplementary material for: Maternal antibiotic treatment affects offspring gastric sensing for umami taste and ghrelin regulation in the pig
Source: J Anim Sci Biotechnol. 2021 Mar 18;12:31. doi: 10.1186/s40104-021-00557-3 (PMC7972225; doi:10.1186/s40104-021-00557-3)
Supplement: Supplementary file 1 — Additional file 1: Supplemental Table 1. Primers information and real-time quantitative PCR conditions used in the trial. Supplemental Table 2. Characteristics of antibodies used for immunostaining. [file 40104_2021_557_MOESM1_ESM.docx]

**Online-Only Data Supplements**

**Supplemental Table 1.** Primers information and real-time quantitative PCR conditions used in the trial.

| **Gene** | **Account number,**  **Ensebl/NCBI** | **Oligo sequence (5'→3')** | | **Amplicon length, bp** | **Annealing T** |
| --- | --- | --- | --- | --- | --- |
| *ATP4A* | NM_214291.1 | Forward  Reverse | GCATATGAGAAGGCCGAGAG  TGGCCGTGAAGTAGTCAGTG | 151 | 57°C |
| *GAST* | NM_001004036.2 | Forward  Reverse | GACTCTGCGCCTATGTCCTG  GCTCTTTGCCCCTGTTGG | 123 | 60°C |
| *GHRL* | NM213807 | Forward  Reverse | ACAGGGGAGACAAGGAAAGG  CTCCGTGCCTTCCACCTC | 130 | 62°C |
| *GNAT1* | XM_021068805.1 | Forward  Reverse | TGTGAAACTGCTGCTTTTGG  GCCAGGATGGACTGTAGTGTG | 150 | 65°C |
| *IL8* | AB057440 | Forward  Reverse | CAAGCAAAAACCCATTCTCC  TTTCTCTGGCAACCCTATGTC | 123 | 61°C |
| *MBOAT4* | NM_001190423.1 | Forward  Reverse | CTGGGCTCTTCAAACTCACC GTCTGCATCAGGGACAAAAC | 125 | 60°C |
| *PCSK1* | NM_214038 | Forward  Reverse | ACAGGGGAGACAAGGAAAGG  TGATGGAGATGGTGTAGATGC | 116 | 51°C |
| *SSTR2* | D21338 | Forward  Reverse | AAGTGGAGGAGACCCCGGAC  CCCAGGATGAAGGCGTAGAT | 194 | 61°C |
| *TAS1R1* | XM_021095259.1 | Forward  Reverse | CCGTGGTATTCTTGACTTGG  GAGCCCAGCATGAGGAAG | 166 | 62°C |
| *TAS1R3* | NM_001113288.1 | Forward  Reverse | CACACAACGGGGAACAAGG  AGTCATAGCAGCAGGAGTGG | 100 | 63°C |
| *TNF* | JF831365.1 | Forward  Reverse | CAATGGCAGAGTGGGTATG  GGACCTGGGAGTAGATGAGG | 109 | 62°C |
| *HMBS2* | DQ845174 | Forward  Reverse | AGGATGGGCAACTCTACCTG  GATGGTGGCCTGCATAGTCT | 83 | 62°C |
| *RPL4* | DQ845176 | Forward  Reverse | CAAGAGTAACTACAACCTTC  GAACTCTACGATGAATCTTC | 122 | 60°C |

*ATP4A*, ATPase H^+^/K^+^ transporting subunit alpha; *GAST*, Gastrin; *GHRL,* Ghrelin and obestatin prepropeptide; *GNAT1*, G protein subunit alpha transducin 1; *IL8*, Interleukin 8; *MBOAT4*, Membrane bound O-acyltransferase domain containing 4; *PCSK1*, Proprotein convertase subtilisin/kexin type 1; *SSTR2*, Somatostatin receptor 2; *TAS1R1*, Taste 1 receptor member 1; *TAS1R3*, Taste 1 receptor member 3; TNF, Tumor necrosis factor; *HMBS2*, Hydroxymethylbilane synthase 2; *RPL4*, Ribosomal protein L4.

**Supplemental Table 2.** Characteristics of antibodies used for immunostaining.

| **Antisera** | **Species** | **Code** | **Dilution** | **Supplier** |
| --- | --- | --- | --- | --- |
| **Primary** |  |  |  |  |
| H^+^/K^+^ ATPase (β subunit) | Mouse | A274 | 1:2000 | Sigma Aldrich |
| Chromogranin A | Mouse | MON9014 | 1:100 | Novo Castra |
| **Secondary** |  |  |  |  |
| Conjugated anti-mouse IgG | Goat | Alexa 594 | 1:800 | Thermofisher Scientific |
| Biotin-conjugated anti-mouse IgG | Goat | BA-9200 | 1:200 | Vector |
